# Curriculum learning for human compositional generalization

**DOI:** 10.1073/pnas.2205582119

**Published:** 2022-10-03

**Authors:** Ronald B. Dekker, Fabian Otto, Christopher Summerfield

**Affiliations:** ^a^Department of Experimental Psychology, University of Oxford, Oxford, OX2 6GG, United Kingdom

**Keywords:** decision-making, learning, generalization, neural network, compositionality

## Abstract

Generalization (or transfer) is the ability to repurpose knowledge in novel settings. It is often asserted that generalization is an important ingredient of human intelligence, but its extent, nature, and determinants have proved controversial. Here, we examine this ability with a paradigm that formalizes the transfer learning problem as one of recomposing existing functions to solve unseen problems. We find that people can generalize compositionally in ways that are elusive for standard neural networks and that human generalization benefits from training regimes in which items are axis aligned and temporally correlated. We describe a neural network model based around a Hebbian gating process that can capture how human generalization benefits from different training curricula. We additionally find that adult humans tend to learn composable functions asynchronously, exhibiting discontinuities in learning that resemble those seen in child development.

## Significance

In novel situations, people need to repurpose past knowledge to guide behavior (generalization). How they do this remains a mystery in cognitive science. Moreover, building machines that can achieve this is a key goal in machine learning research. Here, we designed a paradigm that allowed us to study the circumstances under which people learn and generalize. We find that people generalize to new situations in ways that are not possible for standard neural networks. However, if networks are modified in a simple way, they can display the same sorts of generalization as people and the same costs and benefits from different training curricula. Our results are relevant to understanding how both biological and artificial agents can deal with novelty.

It is often asserted that humans are good at adapting to novelty (1, 2). For evidence of this versatility, we are invited to consider the quotidian activities that most people master as adults. Navigating the local environment, managing household resources, and interacting socially all frequently require flexible responses to novel and unanticipated challenges. Indeed, success at handling novelty has been touted as a key ingredient in human intelligence (3–5) and is frequently counterpointed with the narrowness and rigidity of current Artificial Intelligence (AI) systems (6).

However, while anecdotal evidence of human mental versatility abounds, capturing this phenomenon in the laboratory has proved remarkably challenging. People can of course readily perform complex tasks when offered clear instruction in natural language. However, it is less obvious whether they spontaneously solve new puzzles without verbal instruction. Dealing with unfamiliar situations requires the repurposing of existing knowledge and skills to novel settings, which is called generalization or transfer. The scope and limits of human transfer were the focus of a voluminous literature in cognitive psychology across the 20^th^ century. Many studies asked whether reasoning puzzles (such as how to direct X-rays to destroy a tumor) were easier to solve after encountering problems with an analogous solution (such as how to deploy an army to conquer a fortress). Prior exposure to test items with common structure sometimes improves performance, but appeals to transfer have proved hard to disentangle from more mundane explanations that rely on hints or implicit instructions from the researcher (7, 8). Moreover, the human ability to handle novelty seems to depend on transfer distance: generalization is often successful when old and new problems share physical features (near transfer) but frequently fails when problems share common structure yet are superficially distinct (far transfer). For example, more than 100 years ago, Woodworth and Thorndike (9) showed that after extensive training on judging the area of a rectangle, human participants showed no improvement at judging the area of other shapes. This graded dependence on transfer distance makes it hard to make concrete claims about whether humans are adept at dealing with novelty and has ultimately led to pessimistic claims that far transfer—moments of extrapolative insight that link entirely distinct problems—are vanishingly rare (10).

Part of the reason why the detailed study of human generalization yielded unsatisfying conclusions is that researchers of the 1970s and 1980s lacked a computational language for quantifying their study of transfer learning. A relevant framework has arisen over recent years as transfer learning has become a central topic in machine learning. Machine learning researchers conceive of learning as an optimization process in which the parameters of a function are adjusted until inputs are mapped onto desirable outputs. In the setting known as supervised learning, learning proceeds by mapping inputs to ground truth outputs defined by an external oracle or teacher. After being trained on a task such as object classification, researchers measure network generalization accuracy on new, unseen instances of the trained object classes (11). This requires the network to learn a mapping function that is sufficiently smooth to permit interpolation (or near transfer) to similar but nonidentical training exemplars, and a large literature explores the principles that allow this to occur (12–16).

However, the transfer to wholly novel settings—requiring extrapolation or far transfer—remains an elusive goal in AI research. One promising solution revives an old idea in cognitive science, that thought and action are fundamentally compositional. The hypothesis is that the world is structured so that new tasks can often be solved by combining old ones, and thus systems with an inductive bias to explicitly compose new knowledge and skills from existing building blocks may succeed in novel settings (17, 18). For example, an agent that learns the rhythms of jazz and then to play the harpsichord can combine them to play music by Miles Davis on a 17^th^-century instrument. Composing behaviors in this way has the merit of limitless generativity, which was a fundamental argument for the compositionality of language put forward by early cognitive scientists (19). However, canonical neural network models do not naturally exhibit compositional behavior (17, 20) and instead require architectural innovation (21, 22) or the addition of neurosymbolic features (23) to solve far transfer tasks.

In the current paper, we set out to study the determinants and limits of human compositional generalization and how it might be modeled in a neural network. Participants performed a task that involved learning the mapping between symbolic cues (colored shapes) and spatial locations. We trained people on a subset of the cues and evaluated them on held-out examples. This allowed us to systematically probe the factors that help or hinder transfer, including how the training examples are selected and ordered. We identify conditions under which humans can generalize but neural networks cannot. We then show how it is possible to adapt the neural networks to match human performance, including their sensitivity to different curricula. Our results suggest that human transfer is facilitated by composition: the ability to decompose a problem into distinct factors, which can then be recomposed to solve novel problems. Together, these findings provide a robust description of human compositional generalization and offer a theory that might explain it.

## Results

**Fig. 1.**
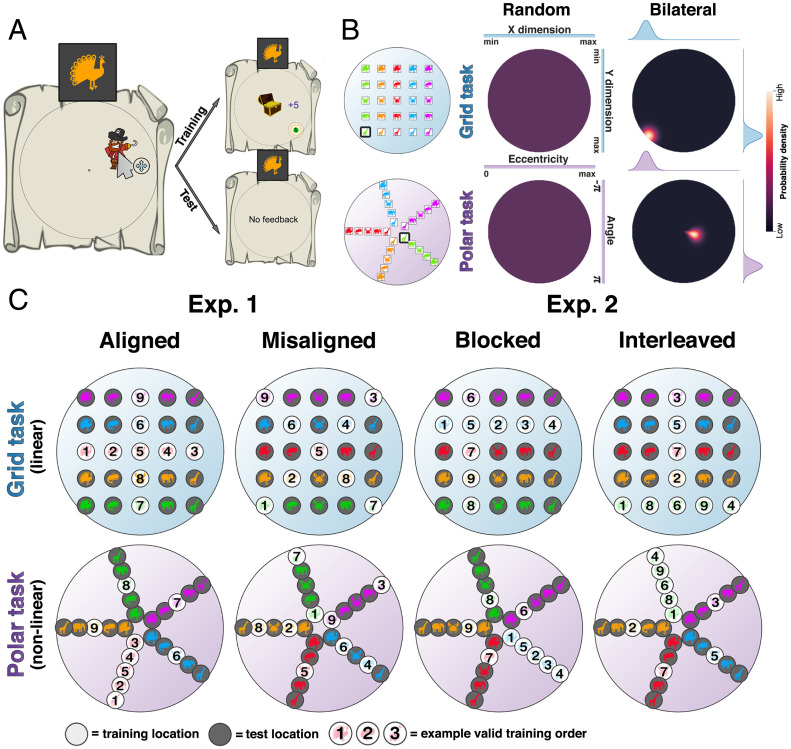
Task and design. (*A*) Participant view. On every trial, the participant receives a symbolic cue from which they must predict the reward location by moving a pirate avatar with the mouse. For training locations, the correct location is then shown, and points are awarded based on proximity. (*B*) Models used for measuring generalization. Under the random model, any response location is equiprobable. Under the bilateral model, response probabilities are modeled as a narrow Gaussian centered on the ground truth (defined in Cartesian coordinates for the grid task and in polar coordinates for the polar task). Our main dependent measure is the LLR of responses under these two models (Eq. **1**). (*C*) *Left*: Exp. 1 design overview. Mapping (grid or polar) was crossed with axis alignment in a 2 × 2 design. In axis-aligned curricula, the training locations are sampled from each dimension while holding the other constant; in axis-misaligned curricula, both dimensions vary at once during training. The ground truth locations for each condition are shown. Superimposed numbers show an example temporal ordering of cues. Training locations are indicated with a white background and test locations are indicated with a gray background for visualization purposes. *Right*: Exp. 2 design overview. Mapping (grid or polar) was crossed with an axis-aligned blocked vs. interleaved curriculum in a 2 × 2 design. In blocked curricula, all instances from a single dimension are trained together; in interleaved curricula, training examples are randomly ordered. Example ground truth locations are displayed in each condition. Superimposed numbers show an example temporal ordering of cues.

Human participants performed a computerized task. They learned to drag a pirate figure to a target screen position within a circular arena in response to a symbolic cue (Fig. 1*A*). Each cue was a colored shape. There were five possible shapes and colors, forming 25 cues in total (Fig. 1*C*). Each participant performed 14 successive blocks; in each block, participants first received supervised training (with feedback) on 9 unique cues (training) and were then evaluated without feedback on the remaining 16 cues (test), each cue being shown exactly once in each block. Importantly, training and test examples occupied distinct locations, so knowledge obtained during training had to be generalized during testing. Between groups, we manipulated the mapping from cue to position. In the grid mapping, each color and shape signaled a ground truth position on horizontal (row) and vertical (column) axes, whereas in the polar mapping, the cues signaled ground truth positions in polar coordinates so that each shape indicated a level of expansion from the center (ring) and each color indicated a degree of rotation (or spoke), or vice versa. We also varied the curriculum specifying which nine cues were chosen for training and their presentation order.

On test trials, generalization required participants to combine information about two spatial factors (horizontal and vertical coordinates in the grid mapping or radius and azimuth in the polar mapping). We predicted that if participants focus on one factor at a time, decomposing the mapping into two functions during training, they will more readily recompose the factors during testing for successful transfer. Thus, in Experiment (Exp.) 1 (*n* = 304), we compared human and network performance as a function of whether training examples were aligned (examples within an axis involved changes in only one feature, e.g., selected from the central row and column in the grid task) or misaligned (selected from the diagonals) to the spatial axes. Our analyses focus on generalization performance among a subset of participants who met a performance criterion during training (*n* = 235; see *Methods* and *SI Appendix* for analysis of training data in the full cohort). For axis-aligned groups, the nine training trials in each block were selected such that first one row/column was sampled and then the other, with the overlapping item always shown in intermediate position 5 (Fig. 1*C*). In other words, we selected all possible shapes of a single color and a single shape in all possible colors as training examples. For axis-misaligned groups, we selected first one diagonal and then the other in a comparable fashion so that shape and color varied together. The logic of this comparison between axis aligned and axis misaligned was that if humans generalize compositionally, they will benefit from a training regime that emphasizes in turn the individual factors from which the state space is composed and thus perform better in axis-aligned conditions. We also applied these curricula to the polar mapping by substituting row and column for ring and spoke.

To allow for fair comparison of errors between conditions, for all analyses, we compared the likelihood of test trial responses under a random model with that under a model describing appropriate ground truth generalization (we are using these models as analytic tools rather than mechanistic theories of how people behave). We call the latter a bilateral model as it assumes generalization using both factors (shape and color), effectively functioning as an oracle, but with Gaussian noise. In the bilateral model, probability mass is a Gaussian distribution centered on the single ground truth location. Under the random model, every possible response location in the arena is equiprobable, modeled by the uniform distribution. Our dependent measure for all analyses is a log-likelihood ratio (LLR):

[1] $$\mathrm{LLR}=\text{log}\left(\frac{p(\mathrm{bilateral}|\mathrm{responses})}{p(\mathrm{random}|\mathrm{responses})}\right)$$

where log() denotes the natural logarithm. A detailed description of these models can be found in *SI Appendix*, Model Fitting (also Fig. 1*C*). When examining test trials using this metric, LLR>0 signals evidence for generalization. We note that under this metric, heuristic strategies such as matching to location associated with the nearest training location do not yield spuriously positive generalization.

**Fig. 2.**
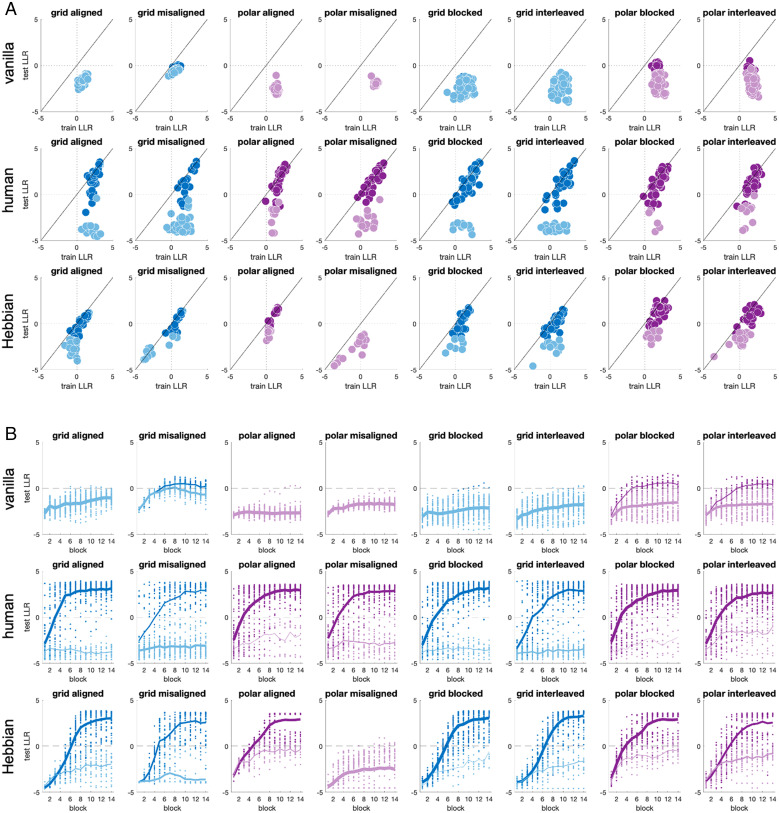
Data from Exp. 1 and Exp. 2. (*A*) Plots of average train vs. test LLR for vanilla neural networks (*Top*), humans (*Middle*), and Hebbian gating networks (*Bottom*; see below). Columns 1 to 4 are from Exp. 1; columns 5 to 8 are from Exp. 2. Each dot is a participant or network colored by whether they were denoted generalizers (dark lines) or not (light lines) for grid (blue) and polar (purple) mappings. (*B*) Learning dynamics, with the test LLR per condition as a function of the experimental block in Exp. 1. Dots are individual participants, and lines are averages of generalizers (dark) or nongeneralizers (light), with line thickness proportional to the number of participants being averaged.

Neural networks received color/shape values as inputs and were trained and evaluated using the same cyclic schedule as humans. The networks were also equipped with output effectors that moved the cursor across the screen by directly mapping output unit activations onto horizontal and vertical translation, rotation, and expansion within the spatial arena. Their resulting performance is summarized in Fig. 2*A* (*Top*, columns 1 to 4), where we plot the average LLR on training trials (*x* axis) vs. test trials (*y* axis). For convenience, we define generalizers as networks (or participants) that were on average better fit by the bilateral than the random model on test trials across the second half of the experiment (mean LLR > 0 on training blocks 8 to 14). Although training LLR values were positive in the later blocks for all 208 networks in all four conditions, the networks generalized poorly. According to our criterion, in the grid mapping, we observed 0/63 generalizers in the aligned curriculum and 24/61 generalizers in the misaligned curriculum, whereas in the polar mapping, we saw no generalizers under either curriculum (Table 1 for full details). The change in performance over time on test trials for standard (vanilla) neural networks is shown in Fig. 2*B* (*Top*, columns 1 to 4). We show the change in performance over training blocks in *SI Appendix*, Fig. S1.

**Table 1.** Statistical summary of empirical results

			Humans		Vanilla neural network		Hebbian gating network	
Exp.	Condition	N	Mean test LLR (SD)	*p* *	Mean test LLR (SD)	*p* *	Mean test LLR (SD)	*p* *
1	Grid aligned	63	91.33 (586.39)	<0.0001	−360.31 (74.11)	1.0000	−126.91 (340.99)	0.0027
	Grid misaligned	61	−381.42 (528.11)		−94.61 (59.37)		−345.31 (490.55)	
	Polar aligned	58	240.08 (415.61)	0.0256	−601.51 (70.54)	1.0000	121.23 (246.04)	<0.0001
	Polar misaligned	56	68.82 (521.11)		−427.61 (41.10)		−645.46 (292.47)	
2	Grid blocked	58	119.10 (499.39)	0.0019	−546.40 (164.18)	0.8937	53.44 (280.44)	0.0156
	Grid interleaved	60	−179.12 (576.63)		−507.05 (176.52)		−65.65 (305.99)	
	Polar blocked	62	270.66 (368.15)	0.0389	−352.15 (223.49)	0.4476	174.91 (261.81)	<0.0001
	Polar interleaved	60	146.52 (383.28)		−357.62 (216.58)		−84.23 (297.74)	

^*^One-sided bootstrap test.

**Fig. 3.**
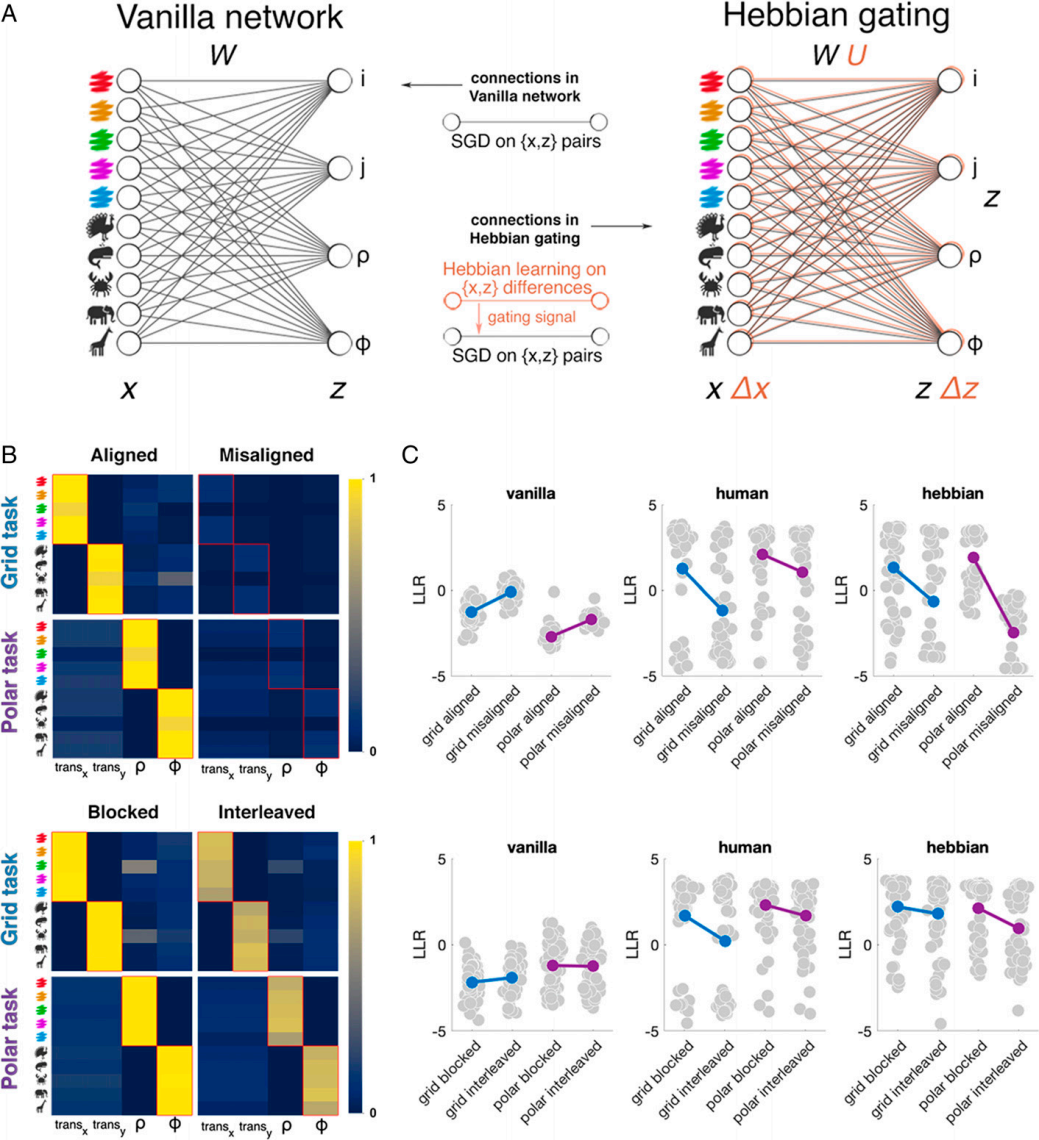
Hebbian gating model. (*A*) Illustration of the vanilla neural network (*Left*) and the Hebbian gating model (*Right*). The Hebbian model consists of a vanilla neural network augmented by a set of Hebbian weights, which act as a gating signal. (*B*) Hebbian weights at convergence in each condition. The plot shows the probability of exceeding the threshold τ at the end of training. The boxes with a red outline are ground truth mappings. *Top*: Exp. 1. *Bottom*: Exp. 2. (*C*) Average LLR on test blocks per condition in Exp. 1 for the vanilla neural network (*Left*), human participants (*Middle*), and the Hebbian gating network (*Right*). Gray dots are single participants or networks. *Top*: Exp. 1. *Bottom*: Exp. 2.

Humans, however, exhibit a very different pattern of behavior. In Fig. 2*A* (*Middle*, columns 1 to 4), we plot LLRs from human training and test trials on Exp. 1. Like neural networks, all human participants converged to LLR > 0 on training trials under all curricula and both grid and polar mappings. This was partly by design: we excluded participants who did not reach a fixed training criterion (see *Methods*). Human generalization performance was heterogenous but was much better than that of neural networks. In all four conditions, human generalization performance (mean test LLR) was greater than that of neural networks (Wilcoxon sign-rank test, all *P* < 0.001). In the grid aligned condition, 47/64 participants were generalizers, whereas only 21/61 participants were generalizers in the grid misaligned condition ($\chi^{2}$ = 20.2, *P* < 0.001). In the polar mapping, performance was better overall, but numerically, more participants generalized under the aligned curriculum (49/58 vs. 39/56; $\chi^{2}$ = 3.6, *P* = 0.06). A nonparametric bootstrap test on LLR values across the cohort also favored the aligned over the misaligned curriculum under both grid mapping (LLR = 91.3 vs. −381.4; *P* < 0.001) and polar mapping (LLR = 240.0 vs. 68.8; *P* < 0.03). Thus, humans generalized better than neural networks, and humans, unlike neural networks, tended to benefit from an axis-aligned selection of training trials. Learning dynamics for humans are shown in Fig. 2*B* (*Middle*); see also Fig. 3*C* and Table 1 for a summary of the findings.

Why did axis-aligned training help? We note that in Exp. 1, axis-aligned and axis-misaligned curricula differ in at least two ways. First, the precise training and testing items are different between conditions. Second, the order in which items occur is different, with the aligned curriculum ensuring that consecutive trials share a dimension value (row, column, spoke, or ring) and the misaligned curriculum ensuring that both spatial dimensions change from trial to trial. To tease these factors apart, next we asked whether a comparable benefit was obtained when stimuli are drawn exclusively from an aligned axis but manipulating whether they were drawn in a temporally correlated fashion, i.e., when cues are blocked rather than interleaved across a dimension (Fig. 1*C*).

In Exp. 2 (*n* = 301; *n* = 242 included for analysis of generalization), we always used axis-aligned training locations but compared two curricula which differed only in the presentation order of the training locations. In the blocked condition, all training locations belonging to one axis were shown before proceeding to the other axis. For example, a participant in the grid task would receive feedback on an entire row and then on an entire column (or vice versa) in any given block. By contrast, in the interleaved condition, training locations were selected from a single row and column but presented in a fully random order. For generality, we sampled training trials from a random ring/spoke or row/column (unlike in Exp. 1, where training trials were always drawn from the central axes). The results for human participants are shown in Fig. 2*A* (*Middle*, columns 5 to 8). Using the same classification criterion (based on LLR in the second half of the experiment), we observed that in the grid mapping, 47/58 human participants were generalizers under blocked conditions but only 36/60 human participants were generalizers under interleaved conditions ($\chi^{2}$ = 6.3, *P* < 0.02). In the polar mapping, we observed more marginal findings based on this binary classification alone, with 57/62 generalizers in polar blocked relative to 48/60 generalizers in polar interleaved conditions ($\chi^{2}$ = 3.6, *P* = 0.06). Bootstrap tests again showed an advantage for blocking in both grid (LLR = 119.1 vs. −179.1; *P* < 0.002) and polar (LLR = 270.7 vs. 146.6; *P* < 0.04) mapping conditions. By contrast, neural networks exhibited no benefit of blocking, and their generalization was overall poorer than that of people (Fig. 2*A,**Top*). Only 1/58 and 13/62 networks generalized in the grid and polar blocked conditions, whereas 0/60 and 14/60 generalized after interleaved training in grid and polar conditions, respectively $\chi^{2}$ < 1.1, *P* > 0.3 in both cases .

We wondered whether these effects might depend in part on participants’ prior assumptions about how the stimuli should be organized in this space. Thus, we collected data on a pre-experimental task in which the 25 cues were placed randomly in a circular arena and participants were asked to arrange them according to their similarity using drag and drop with the mouse. We found that while a grid arrangement was the most popular (observed for 266/476 participants total), we also observed nongrid schemes, including random, circular, and polar arrangements. Participants who created a grid-like arrangement in the pretask had higher test LLRs in the grid task than those who did not, while performance on the polar task was unaffected by grid priors. However, the curriculum effects in both Exp. 1 and Exp. 2 continue to hold even in the subpopulation who created a grid scheme and thus likely already had grid-like priors. As polar arrangements were very rare in the pretask (observed for 8/476 participants), no parallel analysis was carried out for this subpopulation. We describe these findings in detail in *SI Appendix*, Figs. S2 and S3.

### Computational Theory of Curriculum Effects.

Above, we report two main observations. First, most participants can generalize in both grid and polar tasks, whereas (without additional assumptions) neural networks do not. Second, human generalization benefits from both axis-aligned and blocked training curricula. We conjectured that these manipulations allow participants to focus on a single dimension at a time and thus to learn a function that factorizes the two relevant dimensions (e.g., color, horizontal and shape, vertical). Our next goal was to identify the minimal constraints that could be placed on the neural network model to make it display the same pattern of generalization as humans, including sensitivity to curriculum.

Building on both recent (24) and more established (25) findings, we propose a solution that combines error-driven training with Hebbian learning. In this Hebbian gating network, units are connected by two sets of weights (Fig. 3*A*). The feedforward weights W are trained with stochastic gradient descent (SGD) as in the vanilla neural network. The Hebbian weights U (which are separately trained) gate the feedforward weights, so only a subset of connections is active on the forward pass:

[2] $$\hat{z}=Wg_{\tau }(U)x,$$

where x is the two-hot vector of input features; $\hat{z}$ is the output vector of spatial displacements (from the center of the arena) in the four spatial dimensions $\mathrm{tran}s_{x},\mathrm{tran}s_{y},\rho$, and ϕ (horizontal translation, vertical translation, expansion, and rotation); and $g_{\tau }(U)$ binarizes U with respect to a gating threshold τ.

On each training trial t, the Hebbian weights are updated in proportion to the coincidence in discrepancy (or temporal difference) between inputs and outputs. Thus, we define input and output surprise vectors $\Delta x_{i,t}=d(x_{t},x_{i})$ and $\Delta z_{i,t}=1(z_{t}\neq z_{i})$, where $x_{i}$ is a previous trial from the same training block and d(⋅,⋅) is a function that computes the absolute difference (*SI Appendix*, Eq. S3); **1**(*expression*) is an elementwise indicator function, defined to be 1 if the expression is trues and 0 otherwise. We update using a form of Hebbian learning that involves both positive and negative updates, the latter of which is known as preactivated depression (26):

[3] $$U=U+\alpha_{U}\overset{t-1}{\underset{i=1}{\sum }}\left[\frac{\lambda^{t-i}}{\sum_{j=1}^{t-1}\lambda^{t-j}}(\Delta U_{i,t}^{\mathrm{exc}}+\Delta U_{i,t}^{\mathrm{dep}})\right]$$

[4] $$\Delta U_{i,t}^{\mathrm{exc}}=[\Delta x_{i,t}⊗\Delta z_{i,t}]\Delta (1-U)$$

[5] $$\Delta U_{i,t}^{\mathrm{dep}}=[\Delta x_{i,t}⊗1]\Delta U,$$

where [⋅] denotes the dot (elementwise) product, [⊗] denotes the outer product, 1 is the vector [1 1 1 1], and α is a small learning rate. The relative contribution of each training trial i to the total Hebbian weight update is determined by a discount factor λ, resulting in larger updates for comparisons with more recent trials.

The Hebbian gating network is illustrated in Fig. 3*A*, and the convergence values for the Hebbian weights in different curricula are shown in Fig. 3*B*. The feedforward weights W begin by being fully gated. However, gates gradually open up between inputs and outputs that change together. Thus, if, over the course of a block, input $x_{i}$ and output $z_{j}$ (e.g., a color feature and a horizontal location) are consistently changing together from trial to trial, then this Hebbian connection is strengthened, allowing supervised learning to proceed. The model thus implements a simple principle: that by detecting variables that change together over time, we can constrain the solution space for function learning to a small set of generalizable factors.

The Hebbian gating network was able to generalize and to recreate the pattern of successful human performance in grid and polar conditions (Fig. 2 *A* and *B*, *Bottom*). Moreover, using the same set of hyperparameters for all conditions, the model qualitatively reproduced the curriculum effects observed for human learners (Fig. 3*B*), with the exception of the polar misaligned condition from Exp. 1, where the Hebbian gating network generalized more poorly than did humans (Fig. 2 *A* and *B*). Overall, from Exp. 1, 52/63 networks were generalizers in the grid aligned condition, whereas only 32/61 networks were generalizers in the grid misaligned condition ($\chi^{2}$ = 12.8, *P* < 0.001). For polar mapping, the corresponding proportions were 52/58 and 8/56 for aligned and misaligned conditions, respectively ($\chi^{2}$ = 64.9, *P* < 0.001). Similarly, in Exp. 2, there were 47/58 generalizers in the grid blocked condition and 45/60 generalizers in the grid interleaved condition, with 52/62 and 43/60 respectively for the polar mapping; this difference was not significant. However, bootstrap tests revealed an advantage for aligned over misaligned curricula in grid (LLR = −126.9 vs. −345.3; *P* < 0.005) and polar (LLR = 121.2 vs. −645.4; *P* < 0.001) mapping conditions and, similarly, an advantage for blocked over interleaved curricula in both grid (LLR = 53.4 vs. −65.7; *P* < 0.02) and polar (LLR = 174.9 vs. −84.23; *P* < 0.001) mappings.

We also considered other neural network models, including a standard multilayer perceptron, with a hidden layer and outputs that mapped to Cartesian coordinates, and another network which learned separately about shape and color, embedding them in a common hidden layer. None of these alternatives, which are described in *SI Appendix*, Figs. S4 and S5, were able to recreate the observed pattern of human data.

### Asynchronous Generalization.

The theory outlined above proposes that humans (and Hebbian networks) learn to factorize the mapping problem into two subproblems, one for each dimension of the cue. Thus (for example, in the grid task), participants might learn independently that color maps onto horizontal location and shape maps onto vertical location by tracking the coincidence in change among these input and output dimensions. If so, it may be the case that participants’ generalization exhibits an asynchronous trajectory, whereby in between random and bilateral policies, they exhibit a unilateral policy in which a single dimension has been learned. We tested this by fitting a family of models that additionally comprised a unilateral stage, plotting the patterns of errors that participants made sorted by model attribution. As for the analyses above, these were conducted only with participants who met our training criterion (*n* = 478 total across Exp. 1 and Exp. 2).

**Fig. 4.**
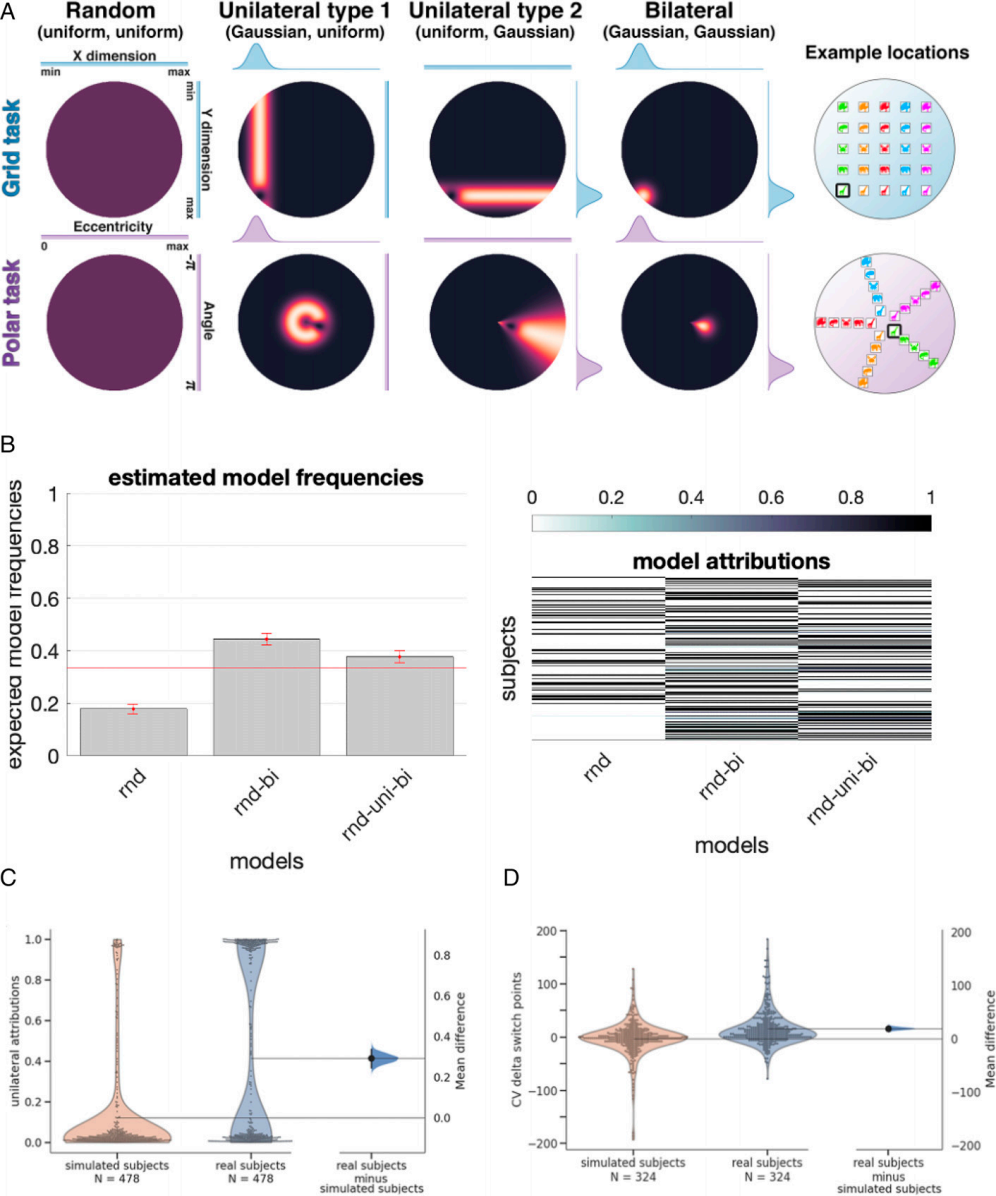
Unilateral errors and modeling setup. (*A*) Probability mass functions under random, unilateral, and bilateral models for an example location (*Right*, highlighted) in the grid task (*Top*) and polar task (*Bottom*). Hot colors are areas of higher probability density. These models were fit to individual subjects, where we allowed switches between models but only in ascending order of number of dimensions learned. Full details on these models are provided in *SI Appendix*, *Model Fitting*. (*B*) *Left*: Expected number of participants best fit by a random model (rnd), a random-bilateral model (rnd-bi), and a random-unilateral-bilateral model (rnd-uni-bi; expected model frequencies from Bayesian model selection). Error bars are 1 standard error of the mean. *Right*: Model attributions (per-model posterior probabilities for each participant). (*C*) Marginal posterior probability of a unilateral stage for each human participant (blue violin plot, *Right*) and simulated participants constructed to follow a rnd-bi policy (orange violin plot). A distribution of bootstrapped differences in means between groups is included, whose black bars represent the 95% confidence interval. *Right:* Cross-validated (CV) difference in per-dimension switch points (i.e., midpoint of a growth curve for the mapping of each dimension to a spatial variable) for simulated and real subjects. Only subjects whose best-fitting model order included the bilateral model were included.

We fit participants’ choices on test trials using models that relied on combinations of random, unilateral, and bilateral generalization policies. Under a unilateral policy, the probability density for a response is consistent with the ground truth for one dimension (e.g., a row) but uniform over the other. Thus, for example, the likelihood of each response is calculated with response to an entire row, column, ring, or spoke, rather than a unique location (Fig. 4*A*). Using Bayesian Model Selection (27), e first compared models that either were completely random throughout or involved multiple consecutive stages, i.e., a random-bilateral or random-unilateral-bilateral model. Across the entire cohort, the random-bilateral model provided the best explanation of the data. However, 38% of participants exhibited the highest posterior probability for a model included a unilateral stage (and only 18% were fit best by a purely random model). We show the expected frequencies and posterior probabilities for each model in Fig. 4*B*. To ask whether a unilateral policy was observed with greater likelihood than chance, we created a simulated cohort whose performance was matched block by block to our participants but whose policy was constrained to lie on the random-to-bilateral axis (see *Methods*). We fit an exhaustive set of models, which involved ascending transitions among these stages—four of which involved a unilateral stage {unilateral, random-unilateral, unilateral-bilateral, random-unilateral-bilateral} and three which did not {random, bilateral, random-bilateral}—to both human and simulated datasets. Over the entire cohort, 277 participants (43%) were fit best by models that comprised an interim unilateral stage, whereas only 75 (16%) of the simulated participants were better fit by unilateral models ($\chi^{2}$ = 183.5, *P* < 0.001). We did not observe differences in the counts of human participants using a unilateral strategy in each mapping or curriculum condition in either Exp. 1 or Exp. 2 (all $\chi^{2}$ < 3.3, all *P* > 0.07). These data are shown in Fig. 4*C*.

**Fig. 5.**
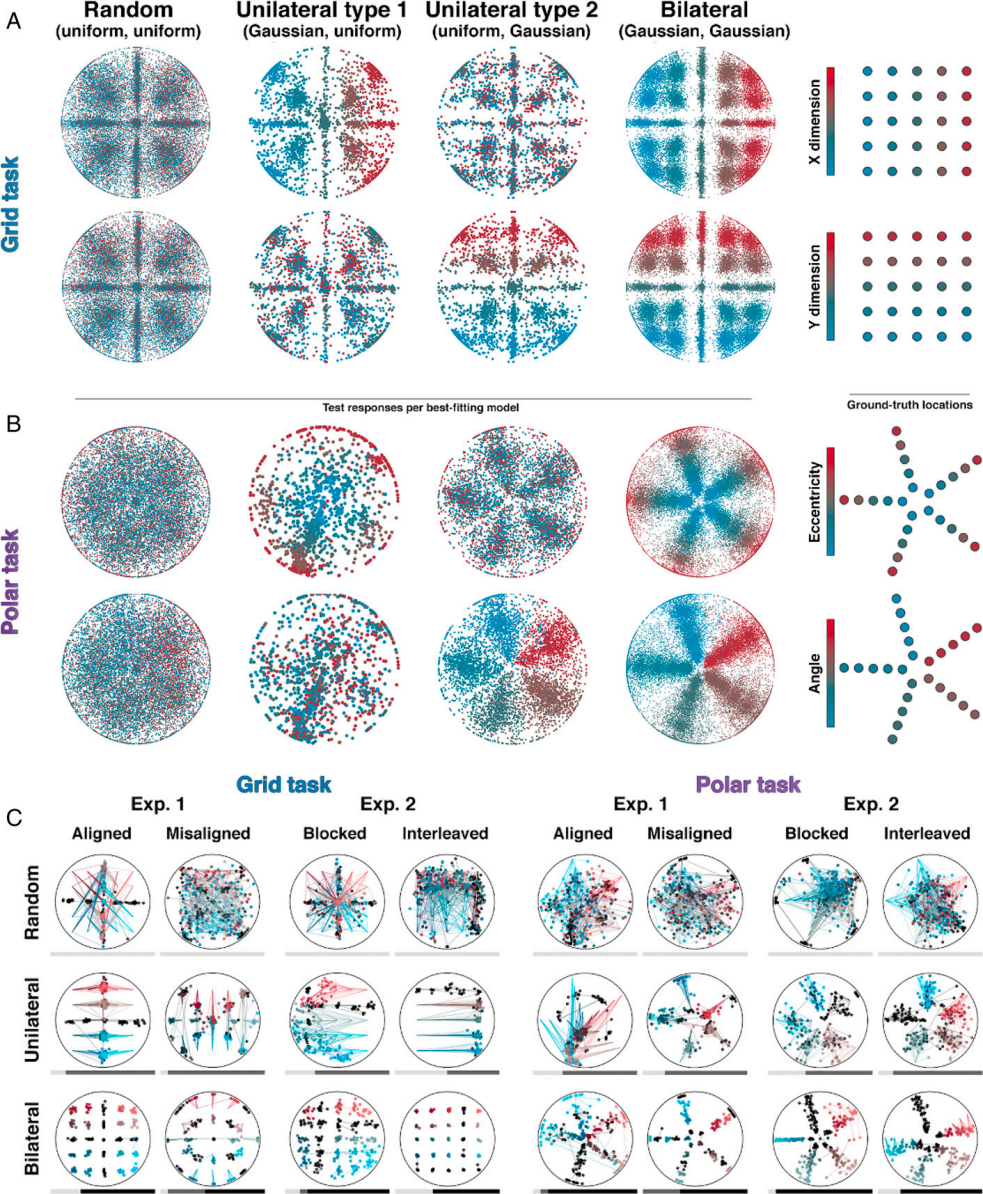
Individual responses under each model. (*A*) Each plot exhaustively shows responses made by participants in blocks classified as random, unilateral (dimension 1 or 2), and bilateral (columns) for the grid task (rows). *Top*: Dots are colored according to the ground truth horizontal translation. *Bottom*: Same responses, but colored according to the ground truth vertical translation. *Right:* Key. (*B*) Similar to *A* but for the polar task. Responses are colored according to ground truth eccentricity (*Top*) or rotation (*Bottom*). (*C*) Example responses from individual participants for different mappings and curricula (columns) and model assignments (rows). Dots are colored according to the ground truth spatial dimension indicated by the symbolic cue (red to blue in dimension 1 and light to dark in dimension 2; black dots are training responses). All responses made in the last stage of the best-fitting model order for the participant in question (e.g., bilateral if the order was random-unilateral-bilateral) are displayed. The bar underneath shows the model assignation over blocks (light gray, random; gray, unilateral; black, bilateral) for the participant in question.

Another way to diagnose the existence of a unilateral stage in behavior is to compute the change in error magnitude for each dimension (horizontal vs. vertical translation and rotation vs. expansion) and to ask whether they decline together across blocks (as predicted by a solely bilateral account) or at different times (as predicted by the unilateral model). Using half of the test data (odd trials), we fit sigmoidal functions separately to error magnitude over time and identified the best-fitting inflection points for dimensions i and j (in the grid mapping conditions) and dimensions ρ and ϕ (in the polar mapping case). This allowed us to order the dimensions into earlier and later for each participant and use the other half of test data (even trials) to compare the fitted inflection point for independently defined earlier and later dimensions. This procedure was then repeated, now using even folds to determine direction and odd folds to determine amounts. The average of both outcomes was then used for each participant. If the errors decline in parallel for each dimension, we expect no consistent difference in switch points between the two dimensions; if there is a unilateral stage, we expect consistent earlier and later attributions for odd and even trials. The latter is what we found. Inflection points were consistently earlier for the early dimension in held-out trials (median = 10 ± 32.5 trials; bootstrap test, *P* < 0.001), and this effect was stronger for participants than in error-matched simulated controls (median = 0 ± 32.9; bootstrap test, *P* < 0.001). We plot the distribution of inflection points, and their differences, in Fig. 4*D*. Finally, in Fig. 5, we plot data from 107,072 responses made by all 478 participants across our experiments, sorted by model attribution, which shows the pattern of unilateral and bilateral responses in both grid and polar mappings (*SI Appendix*, Fig. S6 for the equivalent data divided by curriculum and mapping).

Finally, we explored the temporal relationship between learning and generalization. Did test performance lag behind training, or is learning immediately mobilized for generalization? To test this, we computed ΔLLR, the change in LLR from block to block for training and test trials, and compared them for each of the eight curriculum and mapping conditions across two experiments (we limited this analysis to generalizers). Although, as expected, there was a reliable main effect of the block in each case (as learning saturated over time; all F > 6.8, all *P* < 0.001), there were no interactions between block and condition {train, test} (all F < 2.2, all uncorrected *P* > 0.05). We plot ΔLLR for each block and condition in *SI Appendix*, Fig. S7.

## Discussion

We studied the nature, determinants, and limits of human transfer learning and how it can be modeled with a neural network. We designed a task that required participants to learn and generalize a mapping function from symbolic cues to spatial locations. On test trials, participants were faced with novel, unseen cues but could solve the task by linearly composing previously learned mapping functions. We found that while there was heterogeneity in human generalization, in total, 72% of our participants showed above-chance performance on test blocks from the second half of the experiment. This contrasted sharply with just 11% of vanilla neural networks.

We studied human learning using a supervised task. A large literature has previously studied how humans learn from supervision, with a focus on category learning, in which participants typically learn to discriminate multiattribute stimuli with respect to a bound (28). In this literature, humans learn discrimination boundaries more readily if they are parallel to the input axes, mirroring the benefits of axis-aligned training observed in our paradigm (29). Neuropsychological work has motivated the theory that axis-aligned boundaries permit rule-based categorization, which recruits flexible task representations in the prefrontal cortex, complementing habitual visuomotor association learning in corticostriatal circuits (30). Several neurobiologically informed models of rule-based learning have been proposed, including one that (like our account) relies on a combination of error-driven and Hebbian learning (25). However, it is unclear how readily these models can be adapted to allow rule composition in a transfer learning setting, where choosing the correct response explicitly involves extrapolating beyond the training distribution, which is the challenge that we tackle here. However, it seems plausible that the compositional behavior we observe in human participants relies on the integrity of the prefrontal cortex.

We formalized transfer learning in an uninstructed task that afforded careful control over the relationship between training and transfer. This allowed us to alternate between training and test trials, and thus model the emergence of learning and generalization over time, and to control the mapping and curricula, allowing us to assess how they influenced human transfer. Our results reveal nontrivial human generalization in a laboratory-based experiment. By nontrivial, we mean that 1) the generalization involves extrapolation (via composition), rather than mere interpolation (far, rather than near, transfer); 2) it cannot be described as a demand characteristic of the experiment; and 3) it is a behavior that clearly is not shown by vanilla neural networks.

We note that the vanilla neural networks do not fail to solve the generalization problem because they lack appropriate (or human-like) priors for the task. In fact, we equipped them with a rich set of spatial effectors so that outputs directly mapped onto horizontal and vertical translation, expansion, and rotation within the arena. This distinguishes our work from previous comparisons of humans and neural networks (31). Instead, the vanilla networks failed because there are many possible mappings that solve the training task. In each task (grid and polar), only one of these mappings allows for compositional generalization: in grid, that which privileges horizontal and vertical translation, and in polar, that which privileges expansion and rotation. To successfully generalize, humans must have an inductive bias to infer which pair of spatial effectors is appropriate for each task. This is a bias that is lacking in vanilla neural networks.

Hints as to the nature of this bias were offered by data on how human generalization performance varied with curriculum. During training, manipulations that allowed symbolic features (e.g., red color or crab shape) to be clustered in time—through axis alignment and temporal autocorrelation—consistently improved generalization success. It seems likely that these conditions promote the learning of factorized mapping functions (e.g., color maps to horizontal translation and shape maps to vertical translation) in a disentangled fashion. The idea that learning and generalization require factorization and disentangling is a popular theme in current machine learning (32).

To test this idea, we built a neural network model that used Hebbian learning to gate forward inference. The Hebbian weights were updated when there was a coincidence in temporal difference between input and output features, biasing learning toward input-output connections that covary together within a consistent time window. This principle was sufficient to allow the network to learn and generalize in ways that closely resembled humans and to recreate the effects axis alignment and temporal autocorrelation on generalization success.

The idea draws upon several established themes in psychology and neuroscience. On a computational level, the idea that the brain uses temporal correlations to extract invariant information during sensory representation learning is the basis for slow feature analysis (33) and related approaches based on temporal stabilization (34, 35). However, rather than serving as a principle for local unsupervised representation learning, we assume that temporal correlations help shape a series of gates on the forward pass through the network, acting akin to an attentional filter or control process. Another way of thinking about the computation principle described here is in terms of causal learning and hypothesis testing (36): by learning the relationship between two factors without additional confounding variables, one can identify simple theories that lend themselves to induction. This sort of scientific reasoning was the cornerstone of Piaget’s notion of formal operations and has been argued to be a uniquely human trait (2). Finally, our idea has antecedents in the category learning literature, where temporal autocorrelation of classification rules allows tasks to be learned with minimal interference (37). This phenomenon can similarly be modeled using a Hebbian gating process (24). Indeed, the comparison process here is reminiscent of theories of category learning that rely on encoding similarities and differences between temporally proximal items (38) so that blocking highlights within-category similarity while interleaving highlights between-category differences (39).

We note that the Hebbian network was not a perfect mirror of human performance. It performed more poorly than human participants on misaligned trials both at training and at testing. In part, this is because we excluded humans who failed the training task; in fact, analysis of training data indicated the most exclusions from the misaligned conditions. However, the Hebbian network learned poorly in the misaligned condition where the Hebbian weights remained small (Fig. 4*C*). When faced with multiple, rapidly varying pieces of evidence, people struggle to learn links between the attendant variables, failing to latch on to a hypothesis as the evidence changes quickly. But in its current form, the Hebbian network is prone to overstate these costs. It may well be that people can use other mechanisms, for example, those that rely on hippocampal memory, to bootstrap learning more effectively under misaligned curricula.

An interesting incidental finding was the presence of unilateral errors. It has previously been suggested that human learners attend to a single axis at any given time (40) and that shifts of attention to a single dimension may have theoretical benefits for learning (41). In fact, the idea that humans solve complex problems by breaking them down into simpler parts has a long tradition in cognitive science. During scientific reasoning, people tend to test hypotheses by manipulating one variable at a time (42, 43) and may learn causal associations locally, assuming that variables that change together or share a common cause are necessarily causally linked themselves (44). People are also prone to entertain a single causal model at a time, leading to overly incremental updates to inferred structure (45), a tendency to infer discrimination boundaries one by one (46), or more generally a positive test strategy (47). These phenomena are often attributed to resource rationality, whereby the best possible policy is pursued given limits on cognitive capacity, but our work suggests an alternative: local learning may be beneficial where it allows simple rules to be composed to allow extrapolation away from the training data (48). By contrast, in a vanilla neural network trained from small weights, learning proceeds strictly in parallel across different dimensions: training more efficiently when contexts are interleaved rather than blocked, while the converse applies to humans (37), and failing to show the sorts of extrapolation observed in our human cohort.

Our work establishes that human learners can benefit from temporally segregated training on cleanly dissociable task factors and offers a computational theory that explains this. Nevertheless, there are alternative mechanisms that cannot yet be dismissed. The theory embodied by our Hebbian model is that coincidence in temporal difference allows the gates of learning to open. However, given that our symbolic cues had only two dimensions, we cannot rule out the possibility that people are in fact prone to learn from coincidence in stability (i.e., to form associations between dimensions, both of which do not change) rather than coincidence in change. In our study, this mechanism would have worked equally well. A task involving symbolic cues with three dimensions could be used to arbitrate among these possibilities.

## Materials and Methods

### Participants.

In total, 605 subjects (293 female and 312 male) participated in the studies described here. Participants were recruited on the crowdsourcing platform Prolific and were rewarded £9, plus a performance-based bonus of up to £6. An age range restriction of 18 to 40 y was applied, and participants were required to have a submission approval rate of at least 85% over at least five prior submissions. All experiments were approved by the Medical Sciences Research Ethics Committee of the University of Oxford (approval reference R50750/RE001). Before starting the experiment, informed consent was taken through an online form, and subjects indicated that they understood the goals of the study, how to raise any questions, how their data would be handled, and that they were free to withdraw from the experiment at any time. Data rejection was based on training accuracy in the second half of the experiment. This allowed us to exclude participants in a way that was independent of our main dependent measure (generalization performance). Throughout the experiment, responses were considered to be correct (and positive feedback was awarded) if they were within a Euclidean distance of 60 pixels of the ground truth. As the difficulty of learning the training locations was not necessarily identical between conditions, our rejection criterion was based on the within-condition median absolute deviation, using the default scale constant of *b* = 1.4826 (49), and a rejection threshold of 3 median absolute deviations, described as very conservative in the same source. Application of this criterion resulted in a total of 127 rejections (Table 2). There was no significant difference in number of participants excluded in either grid conditions ($\chi^{2}$ = 0.23, *P* = 0.63) or polar conditions ($\chi^{2}$ = 1.15, *P* = 0.28). The training task was relatively straightforward and involved learning between nine cues and nine locations over 126 trials. We assumed that participants who failed to learn these mappings during training may have been paying less attention to the task, in particular as our data were collected online.

**Table 2.** Exclusion counts per condition

Task	Grid task				Polar task			
Condition	Aligned	Misaligned	Blocked	Interleaved	Aligned	Misaligned	Blocked	Interleaved
Total subjects	73	79	76	74	75	77	76	75
Subjects rejected	13	16	15	16	19	19	16	13

### Procedure.

Trials began with a central fixation point, which was on-screen for 1,000 ms. After this, a large version of the symbolic cue was presented in the center of the screen for 1,000 ms. Then, this was replaced by the circular response area, with an image of a pirate in a randomized starting location. A smaller version of the stimulus was displayed above the response arena for the remainder of the trial. The response arena was a circle with a radius of 265 pixels, delimited by a black perimeter line. A single dot was displayed in the center of the response space as a reference point. Participants responded by dragging the pirate within the arena using the mouse and pressing the *d* button to confirm their response location. If this did not occur within 4 s, a visual timer appeared, which gradually diminished in length. From onset, it took 20 s for this timer to fully deplete. At this point, no response was logged if the pirate was still in the starting position. If the pirate had been moved, its current position was taken as the participant’s response. Upon timeout or response, the pirate disappeared, and a black dot appeared in its place, more precisely indicating the response location. On generalization trials, this lasted 1,600 ms. On feedback trials, this lasted 300 ms, after which an X indicating the ground truth location was added to the display. After another 300 ms, three concentric circles appeared around the X for 1,000 ms, indicating the maximum range at which points would be rewarded. On either trial type, the previous feedback then disappeared and was replaced by the text “no feedback,” the text “incorrect,” or a treasure chest and the number of points earned on that trial, as appropriate. This feedback was displayed for 1,300 ms, after which the trial was concluded. If any trials remained in the current block, this was followed by the fixation stage of the subsequent trial, which served as an intertrial interval.

Points were awarded as follows: 5 points were awarded if the distance between response and ground truth location was less than 20 pixels, 2 points were awarded for a proximity of 20 to 40 pixels, 1 point was awarded for a proximity of 40 to 60 pixels, and 0 points were awarded otherwise. The three concentric circles displayed on feedback trials indicated the ranges at which 5, 2, or 1 points were awarded, and participants could see how many points they earned by which of the three circles was highlighted (or none in the case of no points). The number of points obtained during feedback trials in the current block was displayed on-screen. Participants were instructed that although they would not see how many points they earned during trials for which no feedback was provided, their performance on these trials would contribute toward their bonus payment. The bonus payout was the number of points earned in a block, with a rate of 1 penny per point, up to a maximum of 36 pence per block. In addition, subjects earned a £1 bonus for completing the stimulus dissimilarity rating task prior to the main experiment.

Each experiment consisted of 14 blocks. In each block, each of the 25 symbolic cues was queried exactly once. In the first 9 trials of each block, the training locations were queried, whose order obeyed a condition-specific curriculum, detailed in the next section. In the last 16 trials of each block, test locations were queried in randomized order. Between blocks, a pause screen was shown, displaying the bonus earned by the subject on the previous block and their accumulated total bonus. The participant could continue to the next block when ready by clicking the space button. Mapping of the stimulus dimension onto the rule type and ordinal mapping of the dimension levels to the rule magnitudes were randomized between subjects. In the polar task, the ground truth locations were randomly rotated per subject but were derotated into a common space in our visualizations.

### Experimental Design.

Data from a total of eight (between group) conditions were collected, which are reported here in two experiments. Each experiment crossed a curriculum condition with the (grid or polar) mapping condition in a 2 × 2 between-group design. Exp. 1 tested the effect of axis alignment. In the aligned curriculum, feedback was provided for the central two axes: the central row and column in the grid task or the central ring and one spoke in the polar task. This meant that the set of training locations contained all shape levels for a single color and all color levels for a single shape (e.g., all red stimuli and all crabs). As different stimuli within an axis involved changes in only one rule, we consider the stimulus dimensions and rules to be aligned in this setup. In the misaligned curriculum, the stimulus axes were misaligned with the rule space, forming an X shape in the grid task and an equivalent in the space of the ring/spoke in the polar task. Exp. 2 tested the effect of blocked vs. interleaved presentations. In these experiments, feedback was provided for a random row and column (grid task) or a random ring and spoke (polar task). Again, the first nine trials of each block queried the training locations. In the blocked curricula, trials 1 to 4 queried the locations from one axis, trial 5 queried the location shared between training axes, and trials 6 to 9 queried the locations from the other axis. The axis that was queried first alternated between blocks, with the dimension that was queried first in block 1 randomized between participants. In the interleaved curriculum, training locations were presented in a randomized order. The curricula in Exp. 1 were always blocked. Further information about our model fitting, analysis methods, a pretask performed to measure participant priors, and details of the neural networks used can be found in *SI Appendix*, *SI Methods*.

## Supplementary Material

Supplementary File

## Data Availability

Anonymized human behavior data have been deposited in the Open Science Framework https://osf.io/g8jk5/ (50).
