# Supplementary material for: Curriculum learning for human compositional generalization
Source: Proc Natl Acad Sci U S A. 2022 Oct 3;119(41):e2205582119. doi: 10.1073/pnas.2205582119 (PMC9564093; doi:10.1073/pnas.2205582119)
Supplement: Supplementary File [file pnas.2205582119.sapp.pdf]

Supplementary information for “Curriculum learning for human compositional generalization”

Supplementary methods

Stimulus dissimilarity rating task

The main experiment was preceded by a dissimilarity rating task, aimed at gauging participants’ priors. This was carried out by all participants in the experiment. In this phase, a set of stimuli using colours and shapes (5 levels each) not used in the main experiment were used. Unlike stimuli from the main experiment, they had no grey background. The stimuli from the dissimilarity rating task appeared in random locations in the same circular response space that was used in the main experiment. Participants could drag and rearrange these stimuli. They were instructed to "find a position for each object that someone else would find most intuitive, and easiest to learn". They could continue by clicking a “Finish” button, provided they had moved at least 50% of the stimuli. Clicking the finish button before this point resulted in the message "You'll be able to continue after engaging with the task a bit more". After completing the dissimilarity rating task, participants were advised that there was no connection between the task they had just completed and the main task. Analyses pertaining to the dissimilarity ratings are described in the supplementary materials, under the paragraph ‘Effect of subject priors on learning’.

Cross-validated delta switch point analysis (Figure 4)

To estimate whether dimensions were learned synchronously or asynchronously, *switch points* were estimated for each participant. Switch points were defined as the midpoint of an error curve (over trials) for each dimension of the cue (e.g., the mapping from colour to horizontal translation or shape to vertical translation). These error curves were assumed to be sigmoidal with the following form:

$$e(t_d) = l + \frac{u}{1 + e^{\frac{-(t-b)}{s}}} \qquad \text{(Eq. S1)}$$

where $e(t_d)$ is the error on dimension d and trial t, l and u are lower and upper bounds, and s and b are the slope and inflection point (midpoint) of the sigmoid. We fit this model on odd trials and tested whether the ordering of $b_{d=1}$ and $b_{d=2}$ was consistent when the model was evaluated on even trials. Comparing resulting inflection points on held out even trials resulted in positive differences for consistent differences between folds, and negative values for inconsistent directions between folds, which are plotted in **Fig. 4C**.

Non-unilateral control simulations

In our analyses that test for the presence of a unilateral stage, a set of simulated control participants was created to account for asynchronies in learning that may have occurred due to stochasticity in

responses and stimulus sampling. A simulated counterpart was created for each subject, whose responses were centered on the ground truth with a bivariate Gaussian error term whose covariance was fitted to per-block generalisation accuracy in the corresponding participant. This error distribution was spherical, i.e. with diagonal covariance and equal variances. As a result, for the simulated subjects, higher error rates by definition corresponded to larger errors in both dimensions, whereas real subjects could in principle learn either in parallel or piecewise. Simulated responses were clipped to the closest edge of the response space if the Gaussian error placed the response out of bounds.

Neural network models

Let a task T be a pair (X, Y), where $X = \{x_1, x_2 \dots x_{nx}\}$ and $Y = \{y_1, y_2 \dots y_{ny}\}$are variables that respectively correspond to the inputs and outputs of the task. In the experiments described in this paper, the inputs x are 2-hot encodings of stimulus features (of length 10), while the outputs y are output coordinates (distances from the centre of the arena of length 2). These could be in any coordinate system; for convenience we assume they are Cartesian. In addition, we consider that the learner comes pre-equipped with a toolbox of elementary functions $F = \{f_{1z1}, f_{2,z2}, f_{3,z3}, f_{4,z4}\}$ taking arguments $Z = \{z_1, z_2, z_3, z_4\}$. Rather than directly solving the mapping X→ Y, the learner learns a mapping $X \to Z$, the amount by which to apply each function in F given a certain $x \in X$. The main network is a single weight matrix $W \in R^{nx \times ny}$, so that the predicted function amounts $\hat{z}$ are defined as $\hat{z} = xW$. W is initialised as all-zeros.

For simplicity, we assume that functions in Z are called in a fixed order, compositionally acting on the output of the previous function for a predicted output $\hat{y} = f_4(f_3(f_2(f_1(0, z_1), z_2), z_3), z_4)$. In our task, the elementary functions F are eccentricity, angle, x-translation and y-translations, run in this order (which is equivalent to converting the polar coordinates to Cartesian space, and then adding the grid coordinates). As not to privilege any functions by the choice to represent Y in Cartesian coordinates, separate loss functions were used for the different z to score the difference between y and $\hat{y}$ (translation: Euclidian distance $|y - \hat{y}|$; eccentricity: difference in Euclidean norms $\|y\|_2 - \|\hat{y}\|_2$; angle: cosine similarity $\frac{y \cdot \hat{y}}{\|y\|_2 \cdot \|\hat{y}\|_2}$. The weight matrix W was updated using stochastic gradient descent, with learning rate α_W. Neural networks were yoked with human subjects – that is, for each human subject, a neural network was trained, matched in trial structure. The Y scale was the pixel distance in the human experiments, divided by 90. This concludes the description of the vanilla neural network.

The Hebbian gating model is a modified version of the vanilla network, in which all of the above still applies, with the exception of the forward pass function (Eq. 2). In addition, a Hebbian learning mechanism is added, which is defined by Eq. 3-5 and centers on the inclusion of a second set of weights U. Like W, the Hebbian weight matrix U is initialised as all-zeroes.

The thresholding function g_τ disinhibits a weight $W_{r,c}$ if its corresponding Hebbian weight $U_{r,c}$ exceeds threshold τ after divisive normalisation across inputs, where r and c are simply row and column indices. Specifically:

$$g_\tau(U_{r,c}) = \begin{cases} 1 \; if \; \dfrac{U_{r,c}}{\sum_{k=1}^{nx} U_{r,c}} > \tau \\ 0 \; otherwise \end{cases}$$

(Eq. S2)

The distance function $d(x_t, x_i)$ is a comparison function for which

$$d(x_t, x_i) = \begin{cases} 1 \cdot \mathbf{1}(x_t \neq x_i) \; if \; ||x_t - x_i||_1 = 2 \\ \beta \cdot \mathbf{1}(x_t \neq x_i) \; if \; ||x_t - x_i||_1 = 1 \\ 0 \; otherwise \end{cases}$$

(Eq. S3)

Hyperparameters were fitted at the population level, that is, a single set of hyperparameters was used for all conditions. To model inter-subject variability, we opted not to use a fixed threshold, but instead used a normal distribution with mean μ_τ and standard deviation σ_τ. Given a trial order and τ, the model is fully deterministic, and so may be viewed as a proxy for the different priors or policies that humans bring to the task. The mentioned hyperparameters were then optimised using grid search. For each combination of hyperparameters, 40 iterations were performed for each of the 8 conditions, using randomly generated trial structures obeying the condition-imposed constraints. To reduce stochasticity, thresholds were not randomly sampled, but were defined as the 40 evenly spaced points spanning the middle 79/80 of the percent-point function of the threshold distribution. The mean Euclidian error over iterations was computed for each block. Finally, the set of hyperparameters was selected which minimised the sum of per-block mean differences between humans and. The final set of hyperparameters obtained in this way was:

Table S1: Fitted hyperparameters for Hebbian gating network

parameter	value
α_W	0.3
α_U	0.055
ß	.4.6
λ	.65
μ_τ	.0866
σ_τ	.011

Model fitting

As responses were measured at specific pixels, our probabilistic modelling took place in discrete space. The valid response space was a circle with a radius of 265 pixels. In our models, this was implemented by first evaluating probability distributions in a 530 × 530 square response space, then

masking out locations falling outside the circle, then normalising probability mass by the remaining sum to account for clipping effects and discretisation. We used a 2D meshgrid representing the locations of pixels with respect to the screen center. These values were then divided by 180, so that in the grid task ground truth locations fell on the values {-1, -0.5, 0, 0.5, 1} of their corresponding dimension, and the outer edge of the response space corresponded to a Euclidian norm of 265/180 ≈ 1.472. For the polar task, i and j values in the mesh grid were then converted into polar coordinates (angle and eccentricity). The random model was obtained by assigning the same probability to each valid location, which corresponds to the reciprocal of the total number of locations. The bilateral model was obtained by evaluating a Gaussian distribution separately on the i and j meshgrids (angle and eccentricity for polar), masking, normalising, taking the elementwise product of these unidimensional distributions, and normalising again. To unpack this with an example, in the grid task, a Gaussian for the i-dimension would be computed by evaluating a Gaussian function on the i meshgrid. This was then masked (discarding locations outside of the response circle) and normalised (to make the probability distribution sum to one). We call this a ‘raw unilateral model’. Then, this was multiplied elementwise with a similar distribution evaluated on the j - meshgrid and normalised again, resulting in the bilateral model probability mass function. To ensure unilateral distributions modelled errors rather than correct generalisation with greater tolerance, raw unilateral distributions were multiplied by a validity map which was inversely proportional to the bilateral distribution, resulting in the unilateral model. Means of Gaussians were the ground truth coordinates. Standard deviations corresponded to $1/20^{th}$ of the total space. For i, j and eccentricity, this was .147. For angle, this was $\pi/10$. To allow for occasional deviations from systematicity, a lapse rate of 1% was built into each model by using a weighted average of 99% of the main model and 1% of the random model for each model.

As the identity of the model that best explained the data was not expected to remain the same over the course of learning, we allowed model choice to change over time, but only in ascending direction of dimensions learned, resulting in a maximum of two switches per subject. Only test trials were fitted. In determining model order, switches were constrained to only take place between blocks. The primary reason for this is that a priori, we deemed between-block switch points most plausible, as these encompass all feedback trials. In addition, this constrained assigned models to remain stable for at least 16 trials (the number of test trials per block), reducing the risk of overfitting. BIC was used to penalise the additional complexity entailed by multi-model orders (Eq. S4).

$$BIC = -2\log\big(p(data|model\ order)\big) + N_{switches} \cdot \log(224) \qquad \text{(Eq. S4)}$$

Where 224 is the total number of trials. Subsequently, we used Variational Bayes for model selection (49) as implemented in the VBA toolbox (50) to calculate per-subject model order attributions. The inputs for this analysis were $LL' = -0.5 \cdot BIC$, the complexity-adjusted log-likelihoods of each model order for each subject.

Fig. S1

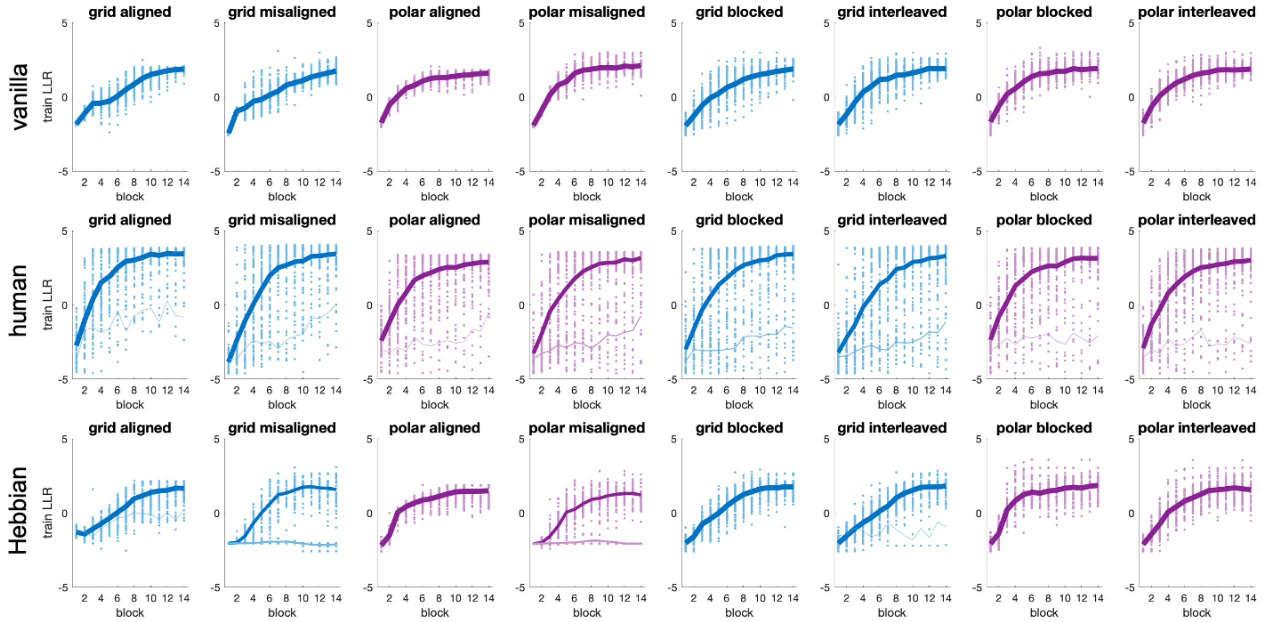

Figure S1. Training data for all human participants (n = 605) and yoked models. *Train LLR per condition as a function of experimental block in Exp.1 and Exp.2. Dots are individual participants, lines are averages of learners (dark) or non-learners (light), with line thickness proportional to the number of participants being averaged.*

Effect of subject priors on learning

A natural question to ask is how the priors that subjects have about the stimulus structure influence subsequent learning. We used a stimulus dissimilarity rating task (for brevity, "arena task" in the remainder of this section) before the experiment to gauge subjects' prior beliefs about the stimulus structure. The arena task was performed by all subjects in the experiment. Specifically, we investigated whether those subjects that created a grid-like arrangement during the arena task were more likely to go on to bilateral generalisation in the grid task than those subjects who did not. (see **Fig. S3** for a visualisation of all subjects' arena responses). No feedback was provided. The used stimuli were still colored animal shapes, but the used colors and shapes differed from the main task, so that it was not possible to match exemplars from the arena task with those from the main task.

We classified patterns of responses using representational similarity analysis (1), obtaining empirical representational dissimilarity matrices (RDMs) from the pairwise Euclidean distances between each pair of stimuli for each subject. Theoretical model RDMs were constructed for grid, non-grid and random response patterns. In the grid model, dissimilarity was lower when either color or shape matched than for pairs where both dimensions differed. We defined two non-grid models around the polar strategy, by taking the average RDM of all possible ground truth arrangements for the polar task. Two versions were used: one in which shape predicted angle and color predicted shape, and one where this was reversed. The non-grid model captured several response patterns in which Euclidean distances were driven to a greater extent by one input dimension than the other, including polar arrangements, clusters based on a single dimension, and structured arrangements of stimuli along the border of the response space (**Fig. S3**). We also defined a random model, in which distances did not depend on the stimulus structure (all distances are equal in the model). The correlation between per-subject empirical RDMs and all model RDMs was computed, and each subject was assigned the model type that had the highest correlation with their empirical RDM. The RDM setup is illustrated in **Fig. S2**. For statistical testing, we discarded subjects best fit by the random model in the arena task and all subjects rejected in the main task (Table 2). Two additional subjects were discarded from this analysis for technical reasons.

We observed that in the grid task, mean test LLRs of subjects with a grid prior (mean=0.25, SD=2.50) were higher than in subjects with a non-grid prior (mean=-1.11, SD=2.55). Mann-Whitney-U=8693, $p<0.001$. In the polar task, we find no differences between the grid prior group (mean=0.81, SD=1.94) and non- grid prior groups (mean=0.82, SD=1.91), Mann-Whitney-U=6720, p=0.904. However, the curriculum effects from the main text are still present when considering only the subpopulation of subjects who were doing the grid task and already had grid priors. Using the same one-sided bootstrap test approach on this subset, mean test LLRs of subjects in the aligned condition (mean=0.83, SD=2.40) were higher than in the misaligned condition (mean=-1.24, SD=2.45), p=0.002. Subjects in the blocked condition also had higher mean test LLRs (mean=1.01, SD=2.10) than subjects in the interleaved condition (mean=-0.11, SD=2.49), p=0.020.

Fig. S2

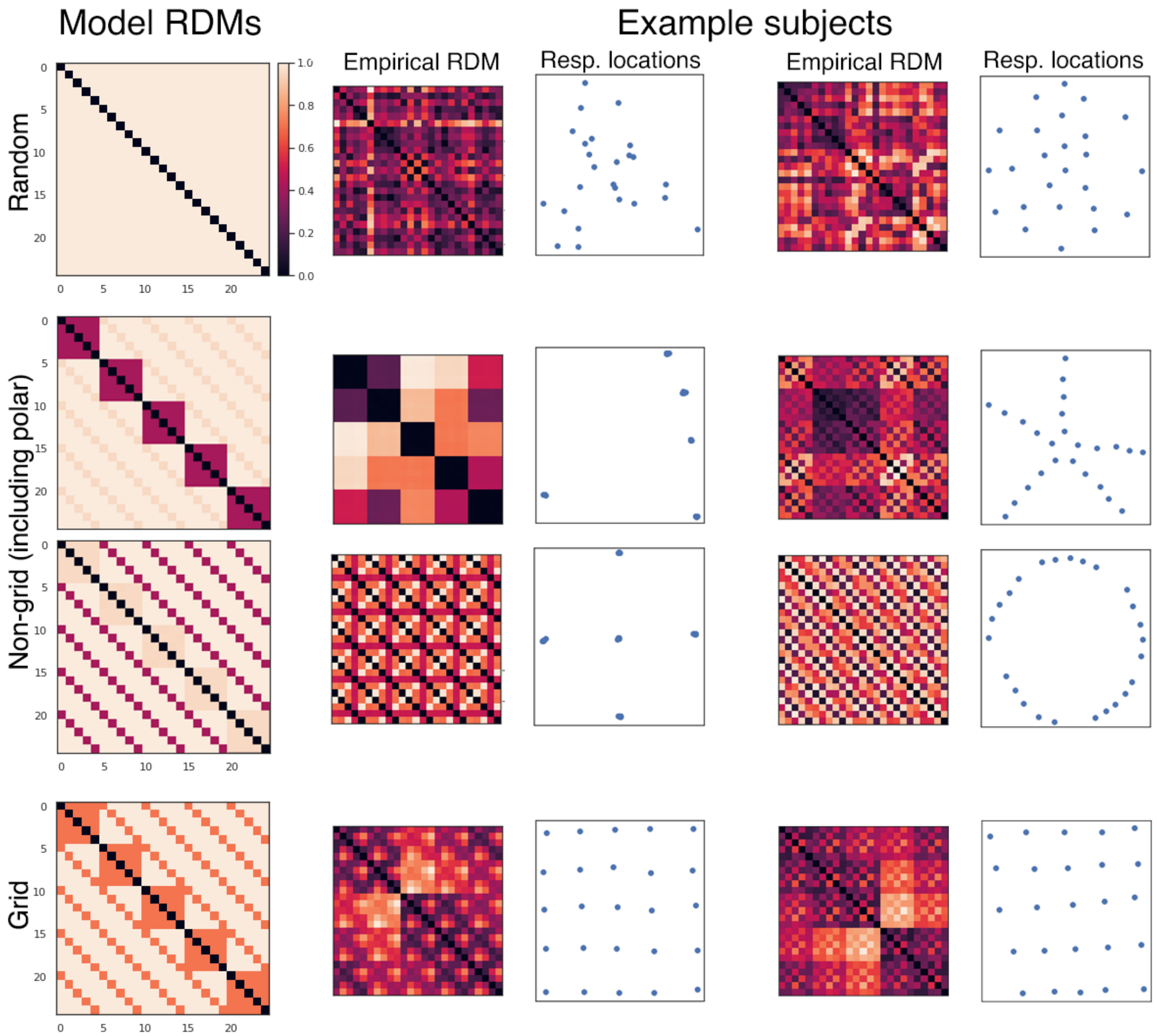

Figure S2: Stimuli dissimilarity rating task analysis. *Left: model RDMs. The grid model corresponds to equal sensitivity to both dimensions (shape and color) in terms of their distances in Cartesian space (Euclidean norms). The non-grid models correspond to greater sensitivity to one dimension than the other. In the random model, distances between stimulus pair are identical, corresponding to a lack of dimension-based structure. Right: Example subjects per assigned model type, with empirical RDM and corresponding response locations.*

Fig. S3

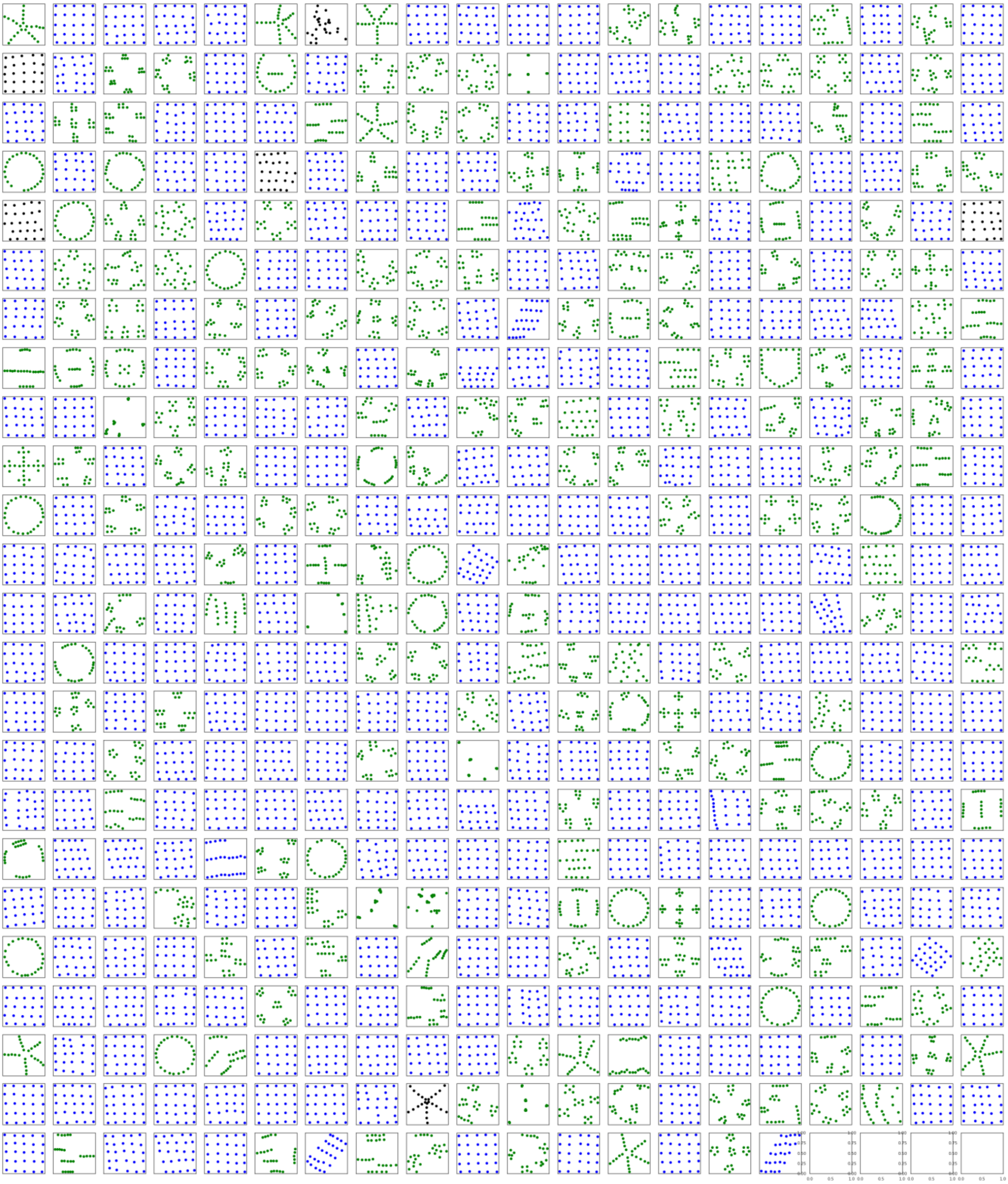

Figure S3: Empirical response patterns in the stimulus dissimilarity rating task. *Each plot shows the responses of a single subject, asked to arrange stimuli composed of shapes and colours not used in the main experiment in a circular response space based on perceived similarity. Responses best fit by the grid model RDM are plotted in blue, non-grid in green and random in black.*

Fig. S4

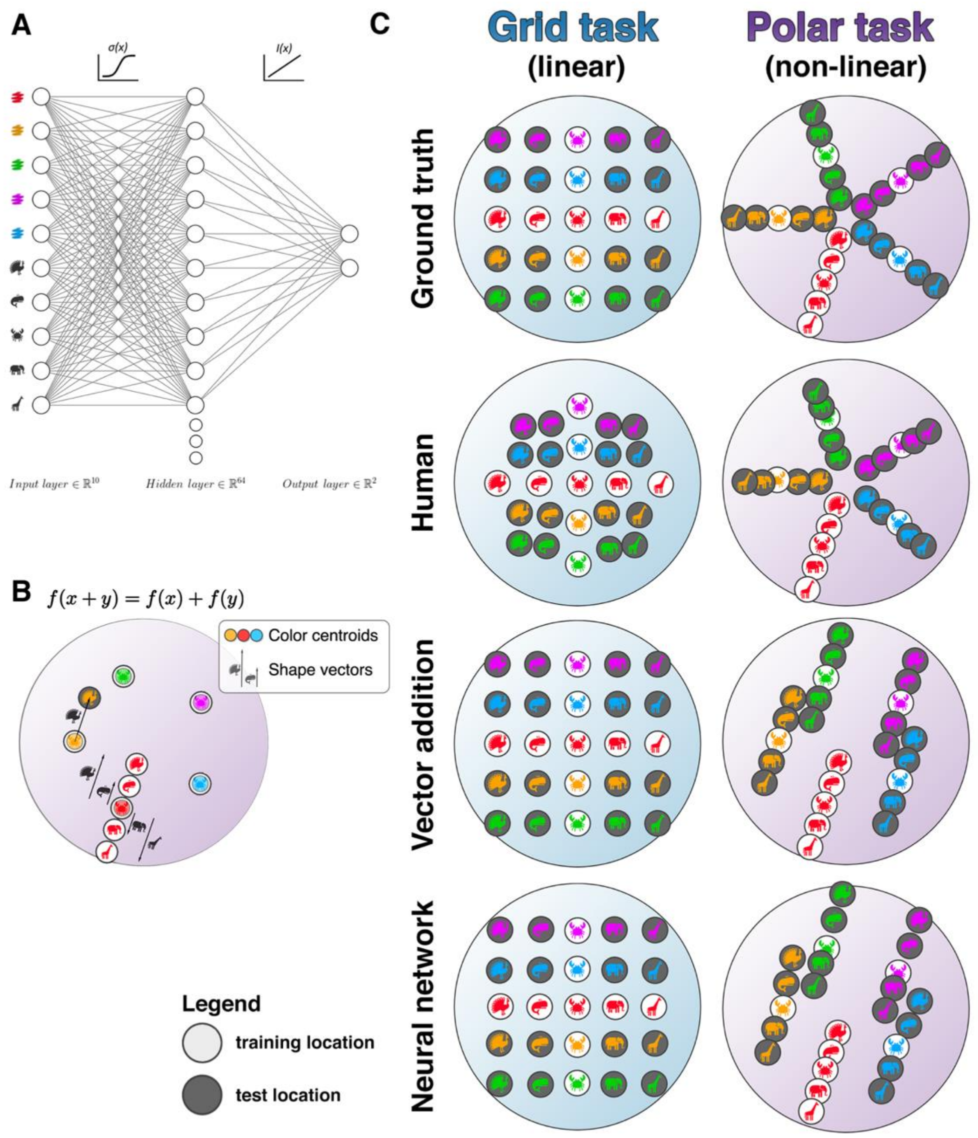

__Figure S4. Comparison to canonical vanilla neural network.__ This network learns to map X→Y without being provided with specialized effectors Z. Not that this is equivalent to learning a mapping X→Z in which Z contains only the effectors $trans_x$ and $trans_y$. (__A__) Neural network architecture. The network is a 1-hidden layer multi-layer perceptron (MLP) with 64 hidden units. σ(x) indicates the sigmoid function and I(x) the identity function (linear layer). To break symmetry, biases and weights were initialised random uniform, with positive and negative extrema of one over the square root of the number of inputs to the corresponding unit. (__B__) Vector addition model. Theoretical model of generalisation in canonical MLP models. We see that neural networks have a "linearity bias", whereby they have a tendency to represent problems as linear transformations when the induction problem is sufficiently under-constrained. This is true by default in their most basic form - linear neural networks — as these are fundamentally incapable of representing nonlinear transformations. In practice, nonlinear activation functions are commonly added to

neural networks to increase their expressivity. Yet even in several popular types of nonlinear neural network, the early dynamics are dominated by the linear solution.

To see why this is the case, consider that networks are typically initialised with small weights (2, 3). In this zero-proximal regime, the hyperbolic tangent approximates the identity function, so that the early dynamics resemble those of a linear neural network. A similar argument can be applied to the sigmoid function, which approximates an affine function in this regime. If the affine solution obtained through these early dynamics fully explains the training data (which is particularly likely when the learner must generalise from few examples), learning stops there, and generalisation is likely to obey the affine solution. The illustration provides an affine solution to our task, and shows how this can lead to incorrect generalisation in the polar task. ***(C)*** *Mean response locations in the second half of the experiment in the aligned condition. Training locations are indicated with a white background and test locations with a grey background for generalisation purposes. Left: grid task. Right: polar task. First row: ground-truth locations. Second row: responses of human participants. Third row: theoretical responses obtained by vector addition model. These were obtained by first calculating the mean position for each colour among training locations, then the vectors required to explain shape differences given a colour, letting the model generalise by adding these two vectors for each shape-colour combination. Fourth row: responses by the MLP network illustrated in A.*

We observe that despite being nonlinear, the behaviour of the MLP closely resembles that of our theoretical model, leading to correct generalisation when the underlying rules can be combined linearly (such as horizontal and vertical translation in a Cartesian space), but not when the underlying rules are non-linear (such as angle and eccentricity in a Cartesian space).

Fig. S5

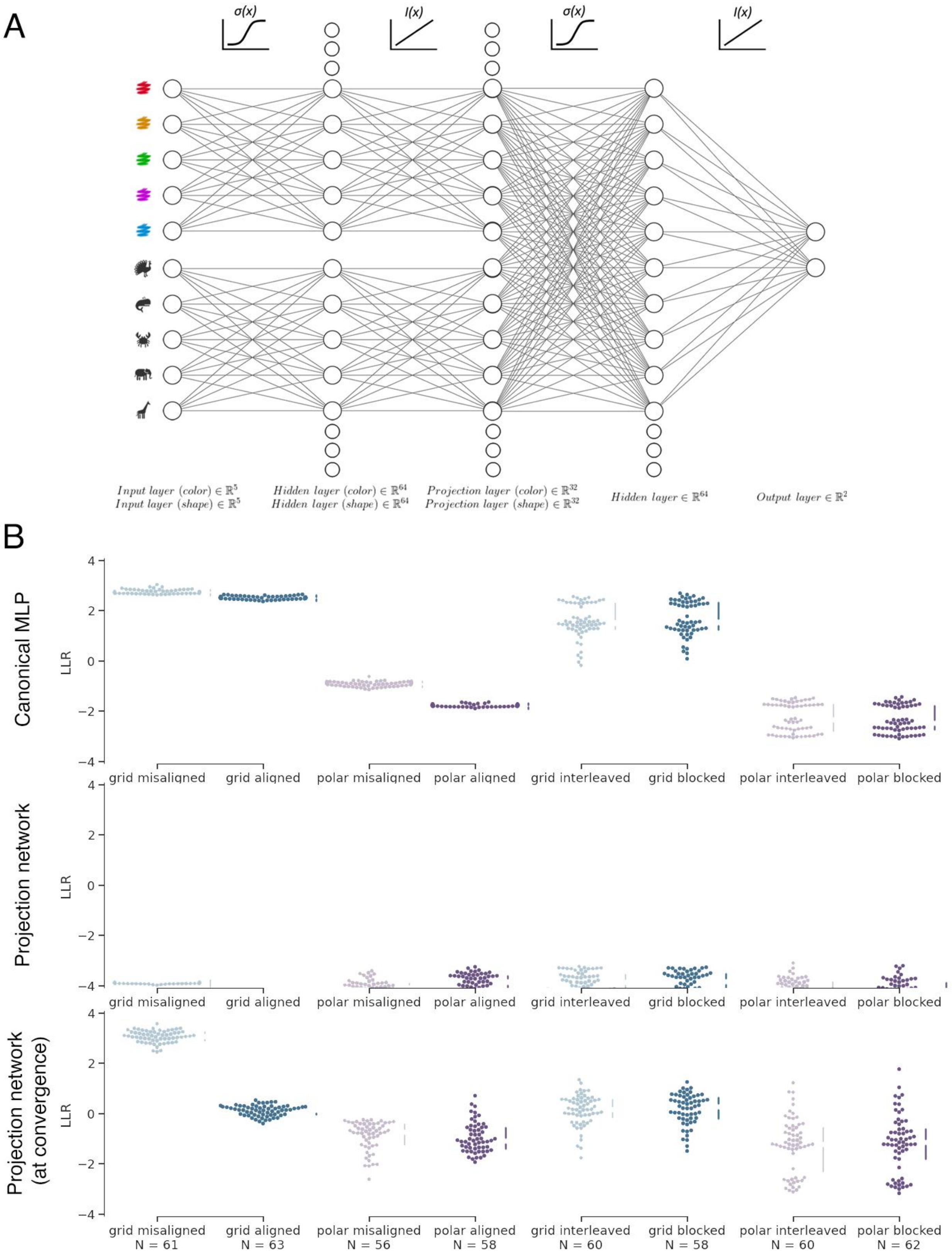

A
σ(x)
I(x)
σ(x)
I(x)
Input layer (color) ∈ ℝ5
Input layer (shape) ∈ ℝ5
Hidden layer (color) ∈ ℝ64
Hidden layer (shape) ∈ ℝ64
Projection layer (color) ∈ ℝ32
Projection layer (shape) ∈ ℝ32
Hidden layer ∈ ℝ64
Output layer ∈ ℝ2
B
Canonical MLP
Projection network
Projection network (at convergence)
LLR
4
2
0
−2
−4
grid misaligned
grid aligned
polar misaligned
polar aligned
grid interleaved
grid blocked
polar interleaved
polar blocked
N = 61
N = 63
N = 56
N = 58
N = 60
N = 58
N = 60
N = 62

Figure S5. Control models. *(A) Projection network. For humans, it should be patent that colour and shape exist as distinct dimensions, but this is not inherently captured by two-hot input encodings. The projection network tests how much is gained by building in knowledge about the input dimension structure. To do this, colour and shape are first processed in parallel onto separate projection layers before computing outputs. Note that this setup is equivalent to one involving 3 MLPs, two for transforming the 5 per-dimension inputs into 32-dimensional projections, and one that takes the concatenated projections (64-dimensional) and transforms them into 2 output coordinates. (**B**) Mean test trial LLRs for different control models. One network was trained for each subject in the human experiment, receiving the same number and order of training and test inputs. Error backpropagation was performed only for training trials. Top row: canonical MLP illustrated in Figure S4A. Middle row: Projection network. Bottom row: Projection network trained to convergence. It is commonly observed that deep networks require more training examples. To allow the more complex projection network to potentially learn the task, we provide an additional control where it is allowed to repeat the experiment 100 times, retaining weights between runs. The last panel displays the projection network's responses in the last of these 100 runs. Tests of curriculum effects matching those used in the main text are provided below in Table S2. The learning rate in each setup was determined by creating a set of 100 valid trial orders for each condition trying each of the learning rates {1e-5, 3e-5, 1e-4, 3e-4, .001, .003, .01, .03, .1, .3} for each model. The learning rate resulting in the lowest mean Euclidian error was then used to calculate model LLRs with trial orders matching human subjects. The resulting learning rates were .03 for the canonical MLP, .3e-5 for the single-run projection network, and .003 for the projection network trained until convergence. Error bars indicate median and quartiles.*

Table S2: Statistical summary of curriculum effects in control models

			Canonical MLP		Projection net (single run)		Projection net (converged)	
Experi-ment	Condition	N	Mean test LLR	*p**	Mean test LLR	*p**	Mean test LLR	*p**
Exp. 1	grid aligned	63	2.51	1.000	-4.51	1.0000	0.11	1.0
	grid misaligned	61	2.75		-3.89		3.06	
	polar aligned	58	1.68	.1842	-3.85	.6554	0.16	.3703
	polar misaligned	56	1.57		-3.82		0.12	
Exp. 2	grid blocked	58	-1.77	1.000	-3.86	.0001	-0.95	.7164
	grid interleaved	60	-.92		-4.08		-0.89	
	polar blocked	62	-2.33	.8039	-4.11	.6176	-1.18	.3802
	polar interleaved	60	-2.25		-4.09		-1.24	

* one-sided bootstrap test

Fig. S6

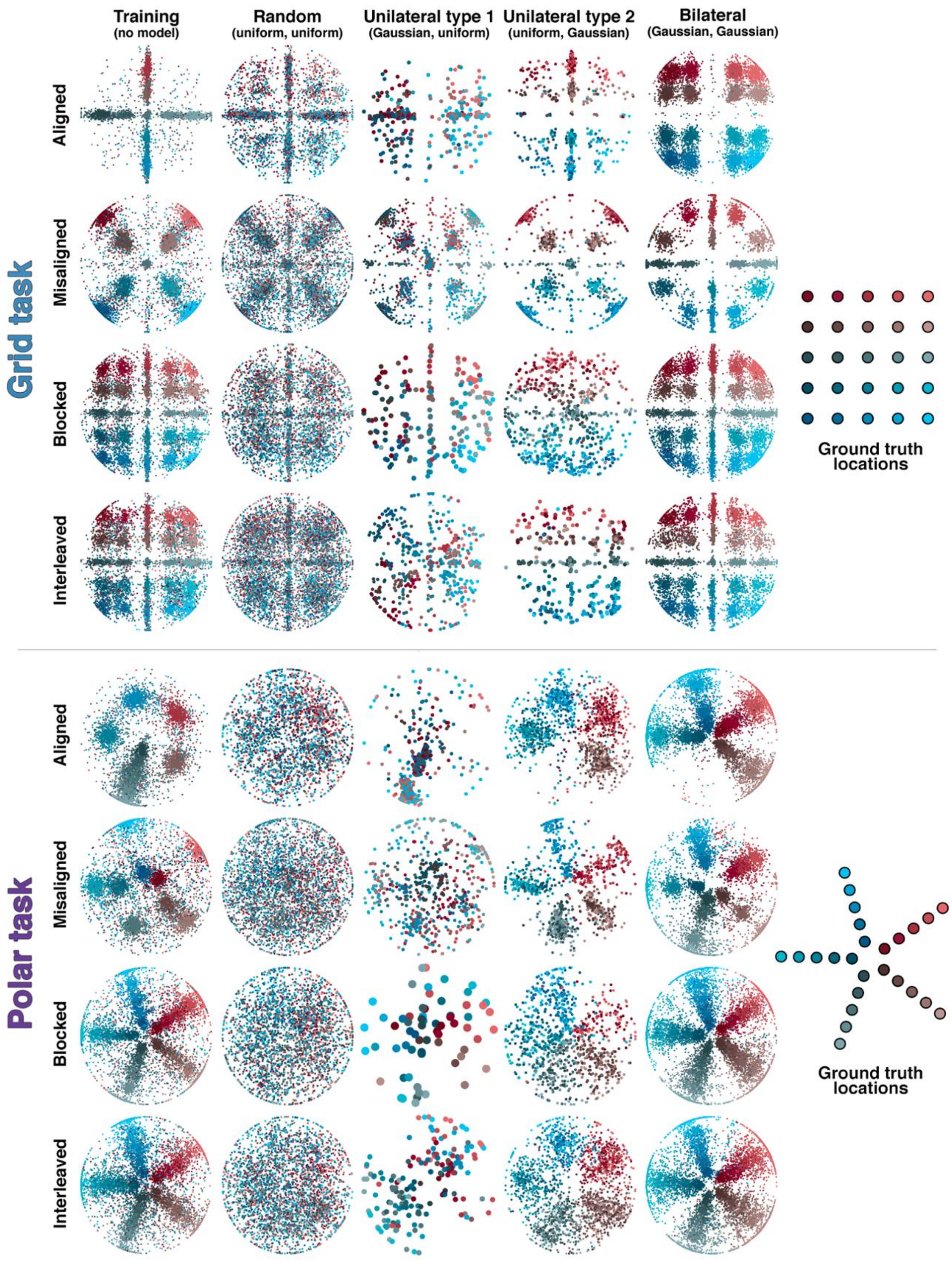

Figure S6. Individual responses in each of the eight conditions. *Plotted are training trials (leftmost column) and test trials (remaining columns, sorted by model attribution for each block) for the grid and polar conditions, sorted by their attribution to random / unilateral / bilateral models. Each dot is a response made by a single participant.*

Fig. S7

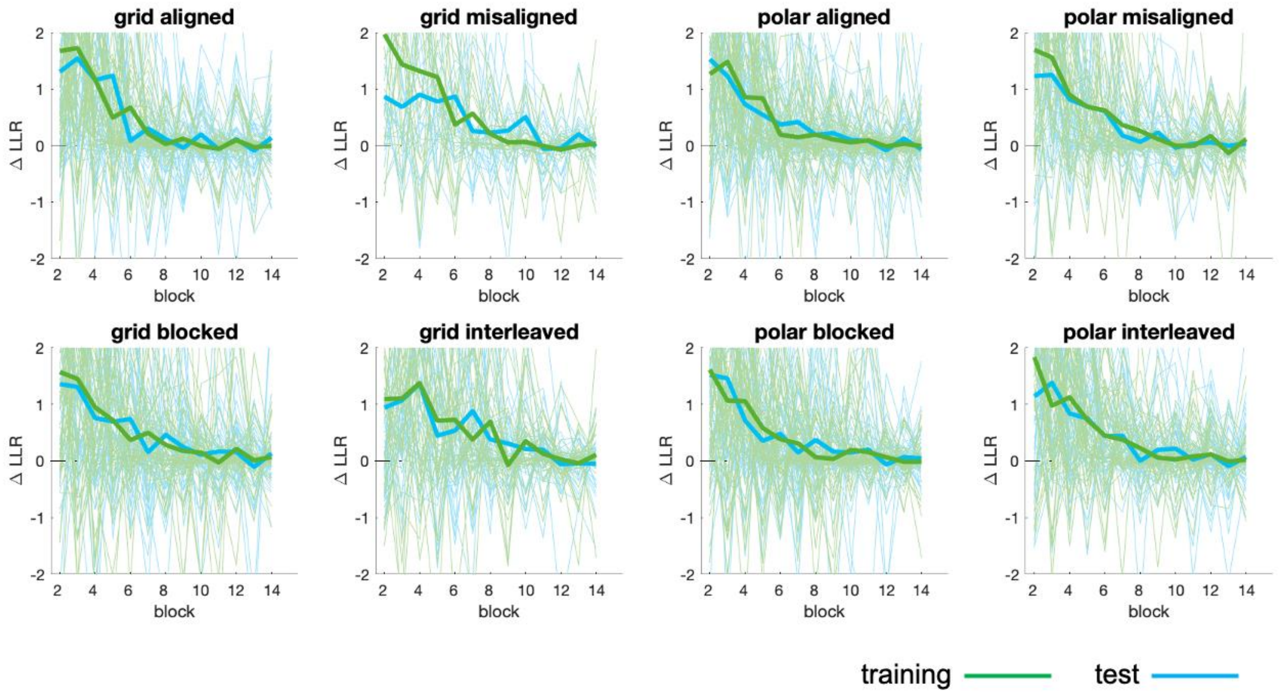

Figure S7. Plot of ΔLLR (change in LLR from block to block) for both training (green) and test (blue trials). *Light lines are individual participants; thick lines are the mean. Training and test tend to overlap, and there are no significant interactions between stage (train, test) and block, implying that learning and generalisation occur in parallel in our task.*

References (supplementary materials)

1. Nili, H., Wingfield, C., Walther, A., Su, L., Marslen-Wilson, W., & Kriegeskorte, N. (2014). A toolbox for representational similarity analysis. *PLoS computational biology*, *10*(4).

2. Glorot, X., & Bengio, Y. (2010, March). Understanding the difficulty of training deep feedforward neural networks. In *Proceedings of the thirteenth international conference on artificial intelligence and statistics* (pp. 249-256). JMLR Workshop and Conference Proceedings.

3. He, K., Zhang, X., Ren, S., & Sun, J. (2015). Delving deep into rectifiers: Surpassing human-level performance on imagenet classification. In *Proceedings of the IEEE international conference on computer vision* (pp. 1026-1034).
